# Humoral immune response and safety of Sars-Cov-2 vaccine in people with multiple sclerosis

**DOI:** 10.1186/s12865-024-00628-w

**Published:** 2024-06-19

**Authors:** Seyedeh Sadigheh Hamzavi, Rosemina Bahrololoom, Sepideh Saeb, Nahid Heydari Marandi, Marzieh Hosseini, Alimohammad Keshtvarz hesam abadi, Marzieh Jamalidoust

**Affiliations:** 1 Department of Pediatrics, Namazi Teaching Hospital, Shiraz University of Medical Sciences, Shiraz, Iran; 2 Alborzi Clinical Microbiology Research Center, Shiraz University of Medical Sciences, Shiraz, 71936-13311 Iran; 3 Department of Virology, Lorestan University of Medical Sciences, Khorramabad, Iran; 4 Department of Biostatistics, Shiraz University of Medical Sciences, Shiraz, Iran

**Keywords:** COVID-19, Disease-modifying therapy, Immunity vaccine, Multiple sclerosis, Vaccine side effects

## Abstract

**Background:**

For the past three years, the pandemic has had a major effect on global public health, mainly on those with underlying medical conditions, such as people living with Multiple Sclerosis. Vaccination among this group is of great importance, and the long-term impacts of vaccination and its safety on the health of these patients will continue to be revealed. Therefore, risks related to vaccination and immune response need to be assessed. The objective here was to characterize the immune response, short-term safety, and the effects of multiple variables on these factors after COVID-19 vaccination (mainly Sinopharm) among people with Multiple Sclerosis. We assessed the short-term safety and humoral SARS-COV-2 anti-RBD IgG response using a data collection form and Immunoassay, respectively.

**Results:**

No severe adverse events or MS relapse was observed. Myalgia/body pain (26.7%), low-grade fever (22.2%), and mild headache (15.6%) were the most common adverse events. The use and type of vaccine influenced the frequency of side effects with a p-value < 0.0001. Regarding immune response, patients on rituximab and fingolimod had a lower antibody titer compared to other medications. With a significant difference, hybrid immunity (p-value: 0.047) and type of DMTs (p-value: 0.017) affected the humoral response.

**Conclusion:**

There is a low incidence of serious adverse effects, MS worsening or relapse after COVID-19 vaccination, and mainly, side effects are similar to that of the general population. It appears that treatment with various disease-modifying therapies does not induce or worsen the post-vaccination side effects, although some, including Rituximab and fingolimod, may affect the immunity induced after vaccination.

**Supplementary Information:**

The online version contains supplementary material available at 10.1186/s12865-024-00628-w.

## Background

Over the last three years, the COVID-19 pandemic has been a major challenge to global health. The effect of the pandemic on those with a preexisting medical condition, such as people living with Multiple Sclerosis (PwMS), has been largely taken into account in developed countries, whereas it has been less explored in countries with additional socioeconomic burdens [1]. Despite the swift response of national and international organizations to provide guidelines, this vulnerable group has required constant reassessment and modification as the pandemic has proceeded. Globally, it is estimated that 2.8 million people have MS, approximately 35.9 people per 100,000 [2]. With an incidence of 6.7 per 100,000 it is a common neurologic disease in Iran [3].

A growing number of studies have identified the clinical characteristics and outcomes of COVID-19 among patients with MS. According to published literature, these Patients do not have an increased risk of SARS-CoV-2 infection or severe COVID-19 disease per se; however, the risk is elevated in the presence of comorbidities, older age, more significant MS-associated disability, progressive disease course, and ongoing treatment with certain disease-modifying therapies (DMT/ DMTs) [4–6]. On the other hand, SARS-CoV-2 infections can be followed by an exacerbation of MS and failure of DMT [7]. Yet the long-term impacts of the pandemic on the health of these patients will continue to be revealed. The widespread administration of vaccines has played an essential role in reducing and controlling the pandemic [8]. In patients with MS, concern has been raised over the effectiveness and the potential adverse effects of vaccinations on different immunomodulators. This being said vaccination, its safety, and adverse effects among this group of people are of significant consideration worldwide.

According to studies, both local (pain, redness, and swelling at the site of the vaccination) and systemic side effects (fever, fatigue, headache, chill, nausea, vomiting, and arthralgia) have been reported in all four types of vaccines assessed (adenovirus vector-based, mRNA, subunit and inactivated) among this specific population [9]. Although there were reports of new de-myelinations and MS relapses occurring after vaccination, the incident of this event was reported to be the same as for those who were not vaccinated [10]. However, Ricardo Alonso et al. demonstrated MS relapse after the first dose of inactivated vaccine in 1.3% of their study population [11].

DMTs mainly act by modulating the immune system in pwMS. Based on literature by Fredrik Piehl, in2021, the currently approved DMTs include Interferons, Glatiramer acetate, oral immunomodulators (Dimethyl fumarate), cell migration modulators (Fingolimod), cell depleting agents (Rituximab, ocrelizumab) [12]. Anti-CD20 B cell-depleting treatments, like ocrelizumab or Rituximab, are expected to hinder the body’s humoral response to vaccinations due to the key role of B cells in antibody development [13]. One of the main focuses in the discussion of DMT is its impact on the response to COVID-19 vaccination among MS patients. Whilst the majority of PwMs will reach an acceptable antibody level after three vaccine doses, multiple studies have indicated that patients on specific DMT display an impairment in the production of antiSARS-CoV-2 antibodies [14–16].

As patients with MS present a unique population concerning immune response, and the fact that there is still a lack of long-term reliable data on the effectiveness and side effects of vaccines, we aim to evaluate the antibody level, short-term safety, side effects, and the effects of multiple variables on them after the COVID-19 vaccine injection mainly the Sinopharm vaccine (inactivated vaccine-BIBP) in this specific population.

## Methodology

### Aim, participants, inclusion, and exclusion criteria

In this cross-sectional study, we aimed to assess the short-term safety and humoral SARS-COV-2 anti-RBD IgG response using a data collection form and Immunoassay, respectively.

Participants were pwMS who visited our laboratory within six weeks of receiving their last COVID-19 vaccination dose from October 2021 to March 2022.

45 women who visited our laboratory during this time, met our inclusion criteria, agreed to do a survey with us, and consented to participate were included in our study. To ensure the immune system had sufficient time to react to the vaccination, we excluded blood samples from patients vaccinated for less than two weeks before sampling.

### Study design, sampling, and data collection

In this study, we assessed the short-term safety and humoral SARS-COV-2 anti-RBD IgG response using a data collection form and Immunoassay, respectively.

We collected the peripheral venous blood samples gathered during each individual’s routine follow-up appointment at our laboratory, eliminating the need for any additional samples. Using Elisa’s test samples were further assessed for their antibody levels to be measured and compared.

A 2-section data collection form (supplementary material, appendix 1) was designed by our research team to obtain the required information from our participants. The first section of the form included queries regarding general socio-demographic criteria, DMTs, the presence of any other underlying disease, the type of vaccine shot, and the frequency of exposure to COVID-19-positive patients. The second part was allocated to assess the adverse effects of vaccination, disease relapse, or sustained neurological worsening after four to six weeks of vaccine injection in MS patients.

### Immunoassay for the detection of anti-SARS-CoV-2 IgG

The Humoral SARS-COV-2 IgG response was assessed at least 14 days after the second dosage of vaccine administration. Immunoassay for the detection of SARS-CoV-2 IgG antibodies was performed using Quanti-SARS-CoV-2 anti-RBD Elisa IgG (PISHTAZTEB DIAGNOSTICS, Tehran, Iran) based on the S1 domain of the spike protein. The test has a sensitivity of 97.1% and a specificity of 100%. Utilizing the designated cut-off point of 5 RU/mL set by the manufacturing company, we reported these IgG levels as positive. This means subjects with antibody levels equal to or higher than 5 RU/mL have acquired an acceptable immune response. In addition, the IgG antibody titer was classified into three groups lower than 4.75, between 4.75 and 10.25, and higher than 10.25 RU/mL.

### Ethics

Ethics approval was obtained from the ethics committee of Shiraz University of Medical Sciences. Ethical approval number: IR.SUMS.REC.1401.695. Participants were briefed on the nature of the study and its voluntary status, and informed consent was obtained.

### Statistical analysis

Data analysis was performed using Medcalc 22. We used mean ± standard deviation (SD) and percentages to describe the numerical and categorical variables. To test for data normality, the Kolmogorov-Smirnov test was utilized. The Fisher’s exact test, Mann-Whitney, and Kruskul Wallis evaluated group differences. The correlative analyses were calculated and reported by the Pearson correlation coefficient test. A p-value less than 0.05 was considered as significant.

## Results

All of our attendees were women of young age (mean age: 37.5 ± 6.51 years old), who were in their relapsing-remitting stage of MS. 36 (80%) were on different DMTs, as shown in Table 1. All subjects had received at least one dose; 42 (93%) had received the second dose after a mean interval of 33.3 (± 13.72) days from the first dose, and 28 (62.2%) had received the third dose of the COVID-19 vaccine with a mean interval of 172.46(± 57.22) days after the second dose. Regarding prior medical conditions, one was a case of heart disease, and one was pregnant and 19 (42.2%) had a history of previous natural COVID-19 infection. The participants were asked if they had regular contact with probable COVID-19 patients; 17 (37.8%) reported frequent contact due to their daily jobs. The basic and clinical characteristics of vaccinated MS patients are presented in Table 1.

**Table 1:** The basic characteristics of participants

Variables	Study population *n* = 45 (%)
**Age**	37.5 ± 6.51
**Female**	45 (100%)
**Disease-modifying therapies**	36(80.0%)
Interferon beta 1-a Dimethyl fumarate Fingolimod Rituximab Glatiramer acetate	12(33.35%) 5(13.9%) 6(16.7%) 12(33.35%) 1(2.7%)
**Vaccine type**	
Sinopharm Other	40(88.9%) 5(11.1%)
**History of previous COVID-19 infection**	19(42.2%)
**Probable COVID-19 exposure**	
Regular contact (Daily) No contact Seldom contact	17(37.8%) 22(48.9%) 6(13.3%)

### Adverse effects and safety of vaccines

**Fig. 1:**
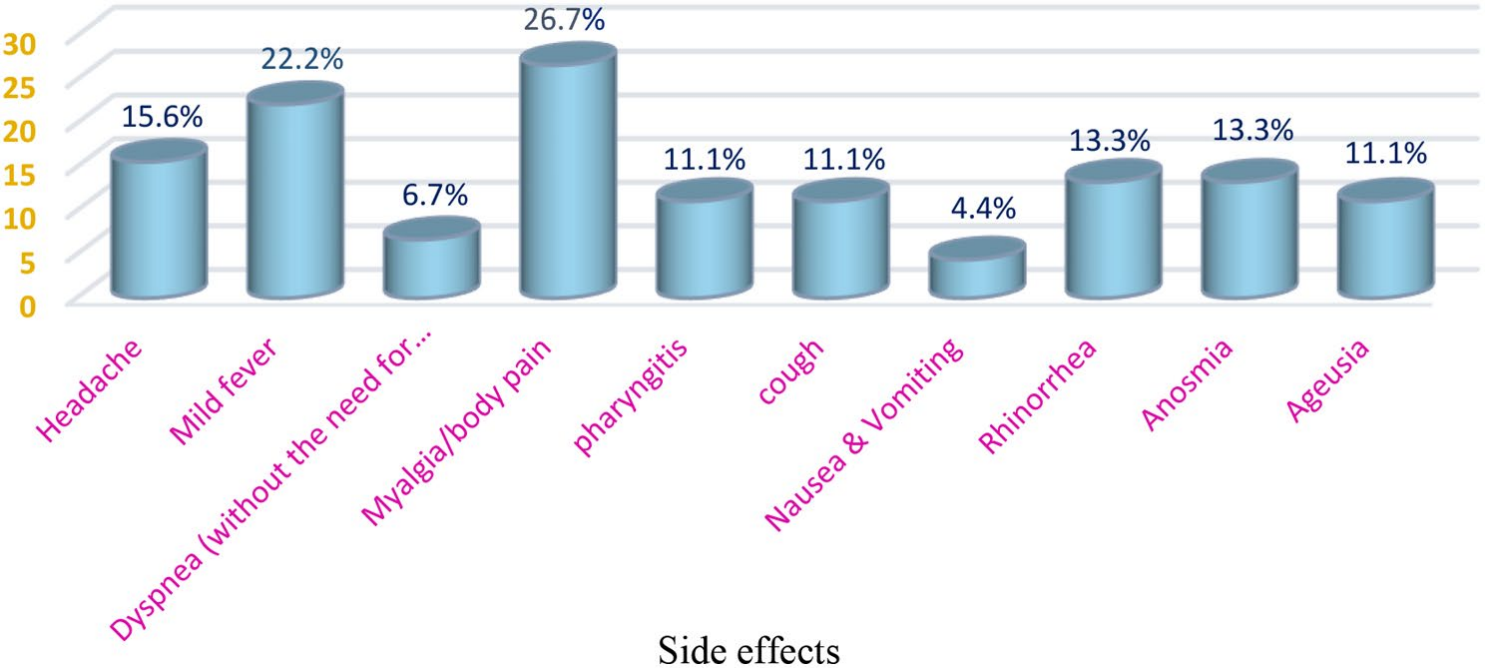
Diversity of post-COVID-19 vaccine side effects. Each bar shows the rate of different symptoms reported by 45 women with MS shortly after COVID-19 vaccination (mostly Sinopharm)

According to our findings, most of our participants did not experience any side effects and of those who did no acute or severe event was reported after vaccination, and they were mainly the side effects reported by people with normal immunity after injection [17]. The list of vaccine side effects reported by MS patients is shown in Fig. 1. The most common adverse events were myalgia/body pain, low-grade fever, and mild headache. No clinical relapse or MS worsening was reported by the time we collected the participants’ sample, which was between six weeks of vaccine administration. No side effects were reported by our two participants with specific conditions.

**Fig. 2:**
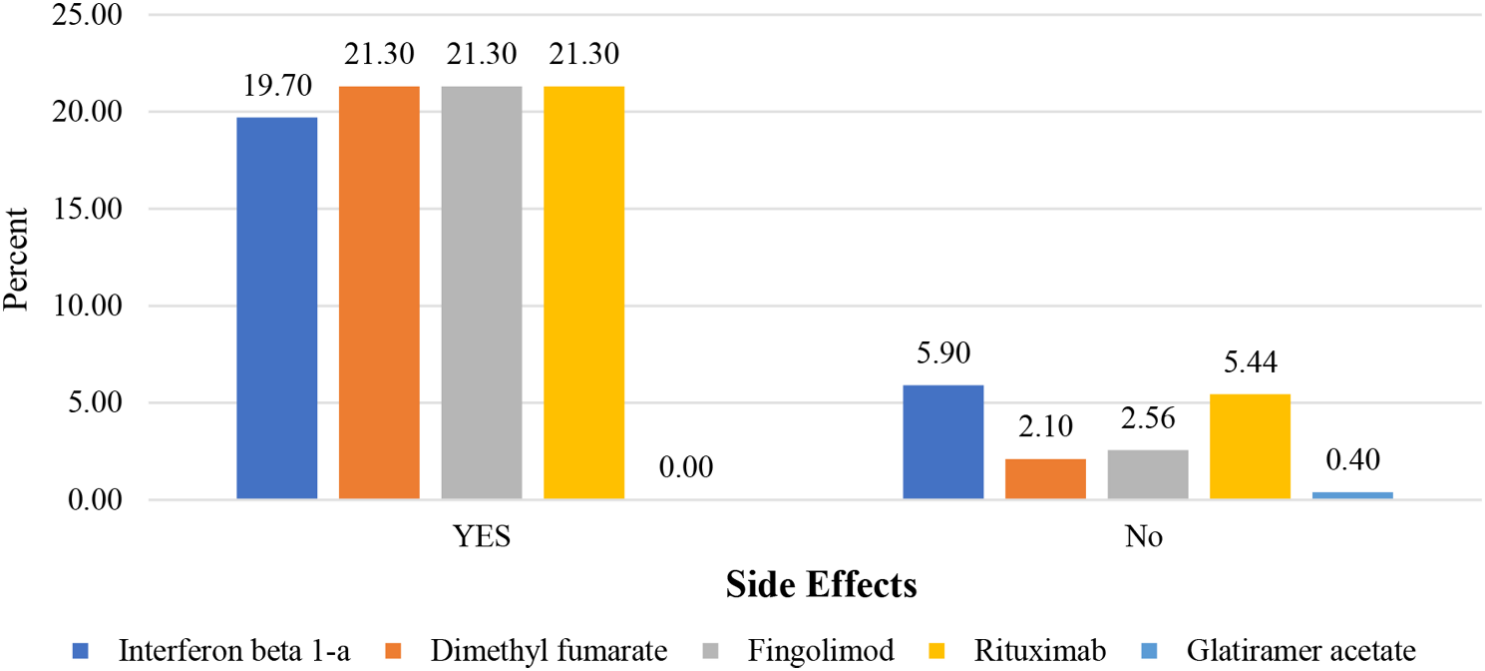
The association between the incidence of post-COVID-19 vaccination side effects and type of DMTs, which met no significant difference (p-value:0.22). The incidence of side effects among MS patients using DMT is broken down by the share attributed to each medication, as shown by the bars. “YES” and “No” are assigned to the side effects’ presence and absence, respectively

**Fig. 3:**
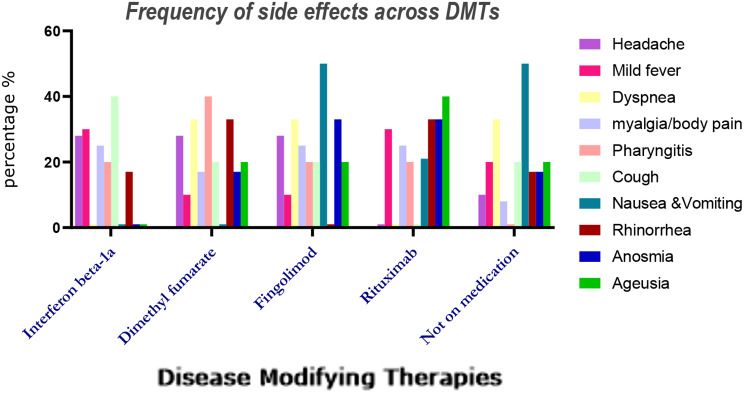
The distribution of short-term diverse post-COVID-19 vaccine (mostly Sinopharm) side effects related to each type of DMT in 45 women with MS.

Among patients who received the Sinopharm vaccine, 14.2% reported experiencing side effects, whereas 8% of those who received other types of vaccines reported side effects (p-value:0.22). Additionally, with a significant difference, those who were on DMTs (83.6%) also presented with a higher rate of side effects compared to those who were not on medication (16.4%). Figure 2 displays the association between the incidence of side effects and different types of DMTs and Fig. 3 describes various type of side effects related to each DMT.

### Immune response

**Fig. 4:**
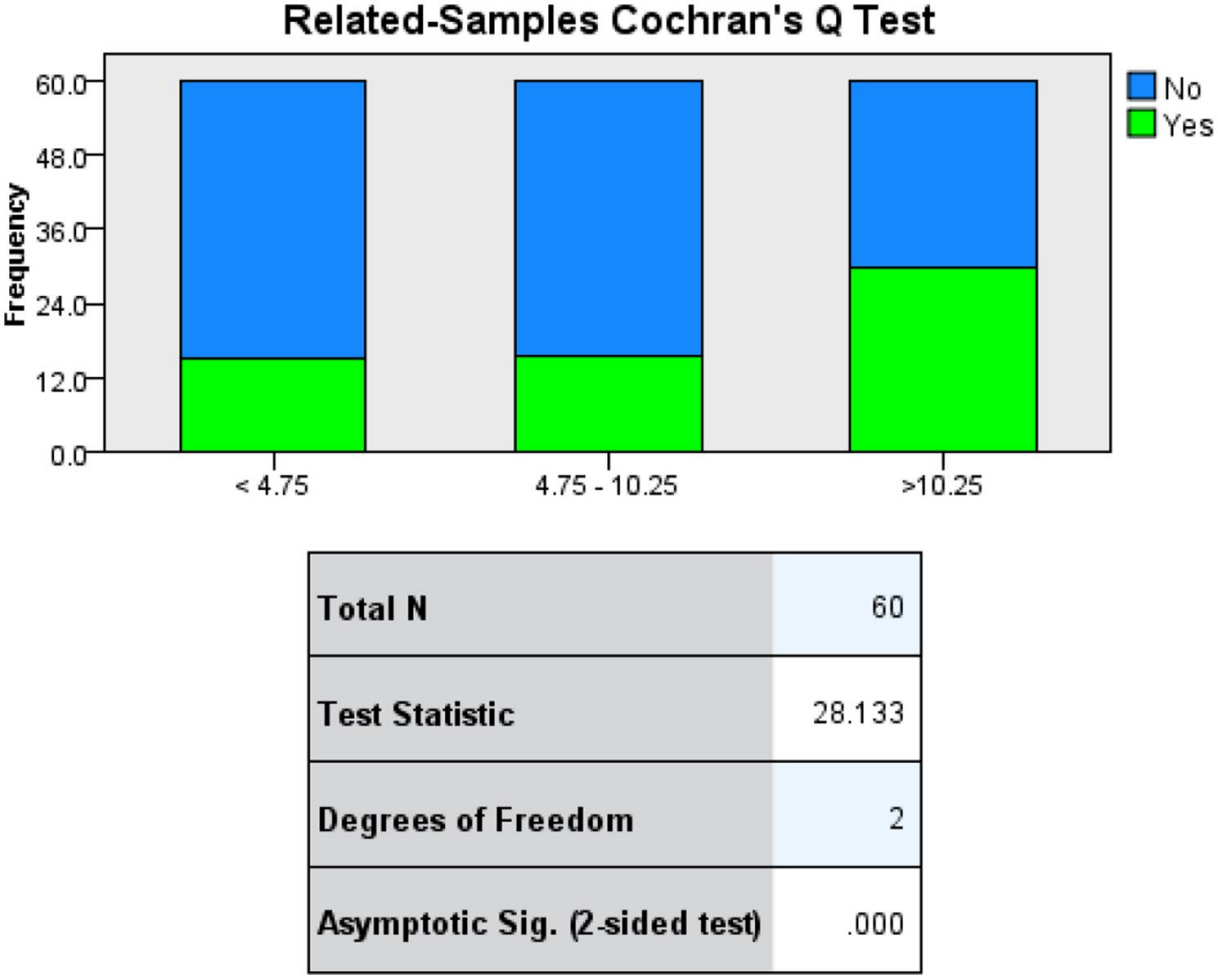
The association between three ranges of antibody levels (< 4.75 RU/ml, 4.75–10.25 RU/ml and > 10.25 RU/ml) and rate of side effects after COVID-19 vaccination (mostly Sinopharm) in 45 women with MS. “YES” and “No” are assigned to the presence and absence of side effects respectively

The highest IgG titer belonged to a patient on interferon beta 1a in her remission phase who had been administered all three doses of the Sinopharm vaccine and was previously infected by COVID-19. The lowest IgG response belonged to two patients on rituximab who had been administered only two doses of vaccines. The relationship between side effects and immune response is depicted in Fig. 4.

The association between immunity induced in different subgroups related to DMT types, doses of vaccination, day intervals from the first to the last dose, previous COVID-19 infection, frequency of exposure, and type of vaccine are reported in Table 2. The same analysis with IgG range classification is displayed in Table 3.

**Table 2:** Percentage of positive immune response (IgG level ≥ 5RU/mL) among 4 groups including numbers of vaccinations, a history of previous COVID-19 infection, different types of DMTs, frequency of exposure, and type of vaccine

Subgroups	Total *n* = 45	IgG ≥ 5RU/mL Positive frequency (%)	IgG<5RU/mL Negative Frequency (%)	*P*-value*
**Vaccination dose**				
One dose	3	2 (66.7%)	1 (33.3%)	0.89
Two doses	14	8 (57.1%)	6 (42.9%)	
Three doses	28	18 (64.3%)	10 (35.7%)	
**Previous COVID-19 infection**				
Yes	19	15 (78.9%)	4 (21.1%)	0.047
No	26	13 (50%)	13 (50%)	
DMTs**				
Patients not on medication	9	6 (66.7%)	3 (33.3%)	
Patients on medication	36	22 (61.1%)	14 (38.9%)	
Interferon beta 1-a	12	10 (83.3%)	2 (16.7%)	0.017
Dimethyl fumarate	5	5 (100%)	0 (0%)	
Fingolimod	6	1 (16.7%)	5 (83.3%)	
Rituximab	12	5 (41.7%)	7 (58.3%)	
Glatiramer acetate	1	1 (100%)	0 (0%)	
**Frequency of Exposure**				
Regular contact (Daily)	17	13 (76.5%)	4 (23.5%)	0.230
No contact	22	11 (50%)	11 (50%)	
Seldom contact	6	4 (66.7%)	2 (33.3%)	
**Vaccine type**				
Sinopharm	40	24 (60%)	16 (40%)	0.635
Other	5	4 (80%)	1 (20%)	

*The comparison of subgroup variables and induced immune response based on chi-square and exact Fisher test, ** Disease-Modifying Therapies

**Table 3:** Range of IgG antibody level induced in 4 subgroups including the numbers of vaccination, history of previous COVID-19 infection, DMT types, frequency of exposure, and type of vaccine. The antibody range is categorized into 3 levels, giving a better insight into a more accurate distribution of IgG titer

Subgroups	Total *n* = 45	≤ 4.75 RU/mL	4.75–10.25 RU/mL	≥ 10.25 RU/mL	*P*-value*
**Vaccination dose**					
One dose	3	1 (33.3%)	2 (66.7%)	0 (0.0%)	0.314
Two doses	14	6 (42.9%)	5 (35.5%)	3 (21.4%)	
Three doses	28	10 (35.7%)	6 (21.4%)	12 (42.9%)	
**Previous COVID-19 infection**					
no	26	13 (50%)	8 (30.8%)	5 (19.2%)	0.043
yes	19	4 (21.1%)	5 (26.3%)	10 (52.6%)	
DMTs**					
Interferon beta 1-a	12	2 (16.7%)	4 (33.3%)	6 (50%)	0.038
Dimethyl fumarate	5	0 (0.0%)	1 (20%)	4 (80%)	
Fingolimod	6	5 (83.3%)	1 (16.7%)	0 (0.0%)	
Rituximab	12	7 (58.3%)	2 (16.7%)	3 (25.0%)	
Glatiramer acetate	1	0 (0.0%)	1 (100%)	0 (0.0%)	
Patients not on medication	9	3 (33.3%)	4(44.4%)	2 (22.2%)	
**Frequency of Exposure**					
Regular contact (Daily)	17	4 (23.5%)	5 (29.4%)	8 (47.1%)	0.466
No contact	22	11 (50.0%)	6 (27.3%)	5 (22.7%)	
Seldom contact	6	2 (33.3%)	2 (33.3%)	2 (33.3%)	
**Vaccine type**					
Sinopharm	40	16 (40.0%)	11 (27.5%)	13 (32.5%)	0.718
other	5	1 (20.0%)	2 (40.0%)	2 (40.0%)	

*The comparison of subgroup variables and 3 range of Antibody levels, based on chi-square and exact Fisher test, ** Disease-Modifying Therapies

The distribution of IgG titer was the same across the frequency of vaccine doses with no significant difference; Although the majority of those with positive Anti-RBD IgG results were part of the three-dose vaccine group. Of those previously infected by COVID-19, 78.9% had developed an immune response (Hybrid immunity); This was 50% for patients without a history of previous infection. The mean interval days from the first to the last vaccination dose were 159 and 136 days for those who tested positive and negative for immune response, respectively, although no significant correlation was observed (p-value: 0.52).

The rate of positive IgG levels was slightly higher in patients who were not on DMTs. As is displayed in Table 2, the highest positive rate of antibodies belonged to patients on Dimethyl fumarate and glatiramer acetate. While the least belonged to patients on Fingolimod with a significant difference (p-value: 0.017). With regards to the optical density, the IgG titer in those with three doses of vaccination was almost 1.77 and 2.78 folds higher than two doses and one dose of vaccine, respectively.

## Discussion

Since Sinopharm was one of the region’s earliest and most available vaccines, and vaccination is essential amidst high-risk communities, most pwMS administered this vaccine at the time. With literature published regarding different vaccine outcomes, inactivated vaccines became known to have fewer side effects than other available vaccines [18–20]. Hence, they became popular among immune-compromised patients. Our data aligned with this assumption, as most of our patients had administered the Sinopharm vaccine, while other vaccines such as Sputnik and AstraZeneca were also available. As a result, it is worth mentioning that the use of adjuvants in Inactivated vaccines, Aluminum hydroxide in the case of Sinopharm, may lead to unfavorable reactions in vaccinated individuals. Both vaccine viral particles and Aluminum hydroxide trigger the immune system [21].

### Short-term adverse effects

As previously mentioned, it was found that the post-vaccine side effect presentations in this particular group were not much different from those of the general population [22]. Most of our subjects didn’t develop any adverse effects. Regardless of the vaccine dose, myalgia, low-grade fever, and headache were the most common vaccine-associated systemic adverse events. Anosmia, rhinorrhea, and ageusia came next in order.

Our data support results from several studies; in one conducted in Saudi Arabia, the side effects of the Sinopharm vaccine were assessed on healthcare workers (HCWs) who had received two doses of the injection. Similarly, they reported myalgia (23.9%), low-grade fever (22.4%), and headache (21%), besides injection site pain and general lethargy, as the top five post-sinopharm side effects [17, 23–25]. In an Italian study, researchers noted the frequency of adverse reactions primarily after administering the second dose of MRNA-based vaccines to individuals with multiple sclerosis. These reactions were consistent with those previously reported and mirrored our findings [26]. Based on a meta-analysis by Sharif et al. in 2021, adverse events were reported in all four types of mRNA: adenovirus vector-based, subunit, and inactivated vaccines [9]. Anosmia and ageusia are among the most bothersome complaint symptoms following COVID-19 infection or vaccination. The incidence was reported to be 3.3 per million in the general population, which is notably higher in comparison to the cases after the vaccination against HPV, meningitis, and influenza during the timeframe of COVID-19 Vaccination [27]. The occurrence of these symptoms was not frequent in previous studies, but our study shows a notable higher rate compared to previous findings.

### DMTs and vaccine side effects

Several investigations have devoted attention to the effect of DMTs on antibody production, whereas few have reported their effects on the development of postvaccine adverse events. In this regard, we observed that post-vaccination side effects were more common in patients on DMTs; Rituximab, fingolimod, and dimethyl fumarate had the same prevalence, while this result was less for interferon beta 1-a and none for glatiramer acetate, however, with no significant difference. Alroughani et al. also reported no significant association between post-MRNA and adenovirus vector-based vaccines and the usage of DMTs on the prevalence of side effects [28].

### Relapse or MS worsening

A particular concern for vaccination of pwMS is the risk of reactivation or disease worsening. While fatigue, facial tingling, dizziness, general weakness, new demyelinations, especially after MRNA vaccination, and MS relapse have been previously reported, overall, the incidence of new symptoms or the exacerbation of symptoms was relatively low [29–32]. Likewise, no relapse or worsening of the disease was observed in our study.

Despite studies indicating a low incidence of serious adverse effects, MS worsening, or relapse after all four types of COVID-19 vaccination, the incident’s likelihood should always be considered. Additionally, it seems that treatment with various DMTs does not induce serious adverse effects. Consequently, we suggest continuing treatment and not pausing or ending it for vaccination.

### Immune response

MS patients’ immunity is dysregulated either due to the disease’s autoimmune nature or as a result of DMTs. This raises concerns regarding the effectiveness and potentiality of vaccines in inducing a sufficient immune response. We investigated the possible influence of different factors on the humoral response, including the number of vaccinations, history of previous COVID-19 infection, different DMTs, the day intervals from the first to the last vaccination dose, and exposure to COVID-19-positive patients. As is displayed in Table 1, interestingly, 66% of those with one dose of vaccine administration had developed a sufficient immune response; this number was slightly less (64%) for those with three doses of administration, although no significant association was observed. This result could be due to the limited number of our participants. Moreover, as anticipated, our findings in Table 2 imply that the IgG titer notably increased with the number of vaccine shots (0.0%, 21.4%, and 42.9% of one, two, and three vaccine doses induced more than 10.25 RU/mL IgG, respectively). Yet statistical significance was not achieved here as well.

In a cohort study by Lustig et al., the Anti-RBD immunoglobulin G (IgG) levels were found to be 1.7 folds higher following the third dose compared to the second dose in the general population [33]. This suggests the efficacy of multiple vaccine injections in the induction of sufficient immune response. The necessity of a booster or the fourth dose should be further investigated. After all, we don’t intend to manipulate the immune system exorbitantly in this autoimmune-associated disease.

### Hybrid immunity

Some studies claim that a COVID-19 infection is as effective as a vaccine dose in providing a sustainable immune response [34]. Besides that, it is known that hybrid immunity, a combination of Sars-Cov-2 infection and vaccination, offers greater protection than natural immunity by itself [35]. Our findings confirm this claim; the rate of positive IgG count was higher in participants with a history of previous COVID-19 infection (hybrid immunity) with a significant difference (0.047).

### Correlation of immune response and adverse effects

Amid the pandemic, this question was raised whether higher rates of post-vaccine side effects point to a stronger immune response. Our study reaffirms the already robust evidence [36]. With a significant difference (p-value < 0.001), an increase in the level of anti-RBD IgG was associated with higher rates of side effects.

### DMTs and immune response

To date, data imply that using different DMTs could influence the development of post-vaccination immunity against SARS-Cov-2. In general, we observed that 62.5% of patients who were not on medication had developed sufficient antibodies. As shown in Table 1, Rituximab (41.7%) and fingolimod (16.7%) have devoted the least positive IgG response to themselves. All Individuals on Dimethyl Fumarate and glatiramer acetate and 83.3% on Interferon beta 1-a achieved a sufficient level of antibody with a significant difference. Concerning the range of antibodies, 80% of patients on dimethyl fumarate had IgG levels higher than 10.25 Ru/mL, which is the highest among DMTs, again with a significant difference. Most studies have demonstrated an adequate response of anti-RBD IgG after vaccination in patients treated with Dimethyl fumarate, glatiramer acetate, and interferon beta1-a [16, 26, 37, 38].

During the pandemic, ocrelizumab and rituximab have been linked to an increased likelihood of severe COVID-19 infection and along with Fingolimod, are the top reported DMTs to cause significant decreases in humoral response after any type of vaccination, although cellular immunity is preserved [39, 40] [16, 26, 38, 40–46]. To delve deeper into the specifics we highlight.

To delve deeper into the specifics we highlight that in autoimmune disease, Rituximab, a monoclonal anti-CD20 antibody, affects the immune system by transitional depletion of B cells while cellular immunity is preserved [39, 47]. Fingolimod, a l-phosphate receptor modulator, acts by regulating lymphocyte egress from lymphoid tissues into circulation [22]. Humoral immunity plays an important role in the success of vaccination in achieving protection against infectious diseases. It seems that due to the mechanism of action of these medications, the immune system lacks proper protection against infection and ends up in the failure of vaccine efficacy. Fingolimod was reported to have the same effect on the antibody level after influenza vaccination [13]. According to evidence, the same effects were expected after COVID-19 vaccination. Therefore Provision of evidence-based information indicating the demand for a booster or fourth dose to MS patients using these medications is encouraged.

### Duration between vaccine doses and immune response

With no significant difference, the mean interval days from the first to the last vaccination dose were higher in patients who tested positive for antibodies compared to those who tested negative. Related literature demonstrated that secondary boosting resulted in a greater enhancement of neutralizing antibody responses when extended time intervals were applied either by vaccination or infection [48, 49]. Although daily exposure to COVID-19 and administered vaccines other than Sinopharm gained a higher antibody titer, statistically, in our study, there was no correlation between these variables.

As a whole, based on previous studies and our findings, it was demonstrated that some elements could influence the vaccine efficacy regardless of vaccine type, including the usage and type of DMT, meantime between vaccine injections, number of vaccine shots, and hybrid immunity. Regarding statistical analysis, some elements were found not to have a significant difference in our study, possibly due to our limitations.

### Limitations of the study

The results of this study should be interpreted in light of its limitations. First, the vaccine’s adverse effects were mostly self-reported and focused on systemic reactions, so we could not empirically confirm their presence. This may cause a recall bias. In addition, according to previous studies, local side effects are the most reported side effects in both MS and the general population. Second, the adverse effects were evaluated after the last vaccine injection dose, which could be the second or the third dose. We did not evaluate whether there was a correlation or difference between the type and severity of side effects after the second or the third dose. Third, this was a single-center study; the number of participants, their gender, and the type of vaccine were limited. This could cause bias in our statistical calculations and analysis. Moreover, the extent to which our findings can be generalized to bigger groups of people and other types of COVID-19 vaccines is uncertain. Fourth, we did not have a base antibody level of our participants to precisely see how much the factors we investigated influenced the level of anti-RBD IgG. Fifth, regarding antibody response, the immunity gained by vaccination depends on both humoral and cellular immunity. We have only investigated the anti-RBD IgG response, which cannot be representative of the exact vaccine-associated induced immunity.

## Conclusion

In conclusion, based on previous studies and our findings, it was demonstrated that there is a low incidence of serious adverse effects, MS worsening or relapse after COVID-19 vaccination, and mainly, side effects are similar to that of the general population. Additionally, it seems that treatment with various DMTs does not induce or worsen the post-vaccination side effects, although some (Rituximab, fingolimod) may affect the immunity induced after vaccination. Some elements could influence vaccine efficacy regardless of vaccine type, including the usage and type of DMT, the mean time between vaccine injections, the number of vaccine shots, and hybrid immunity. Our results could be used to inform PwMS about the likelihood of side effects and could be a background to further studies investigating multiple aspects affecting MS patients’ immunity in the context of similar situations.

### Electronic supplementary material

Below is the link to the electronic supplementary material.

Supplementary Material 1

## Data Availability

The data regarding our participants’ basic and required information and the result of their Antibody titer are available from mjamalidoust@sums.ac.ir.

## Abbreviations

PwMS: people living with Multiple Sclerosis
DMT/ DMTs: disease-modifying therapies
SD: standard deviation
HCWs: healthcare workers
