# Supplementary material for: Humoral immune response and safety of Sars-Cov-2 vaccine in people with multiple sclerosis
Source: BMC Immunol. 2024 Jun 19;25:35. doi: 10.1186/s12865-024-00628-w (PMC11186195; doi:10.1186/s12865-024-00628-w)
Supplement: Supplementary file 1 — Supplementary Material 1 [file 12865_2024_628_MOESM1_ESM.docx]

| **Date …/…/ …..**  **Appendix 1. immune status induced against SARSCoV2 virus and short term safety in Iranian MS affected patients’ data collection form**  **Patient visit Number *:** | |
| --- | --- |
| ............................................ **Occupation:** | |
| **AGE:** ...............  **Year:** ...............  **Smoker? ..............** If yes, cigarettes?.............. Hookah? | **female 🞎 male 🞎 Gender:** |
| **Type of medication:** | **.Stage of MS:** .................. |
| **Have you been vaccinated?**  🞎Yes 🞎No    Date of first dose injection ...............................................  Date of second dose injection ...........................................  Date of third dose injection ........................................... | **Type of vaccine**:  🞎AstraZeneca Bharat 🞎Sputnik 🞎  🞎Sinopharm Barekat 🞎 Pfizer biontech 🞎 |
| **Have you ever tested positive for COVID-19?**  🞎 Yes, Test date ......................  🞎 No | |
| **Exposure to Covid-19 patients:**  🞎 daily exposure  🞎 occasional exposure  🞎 no exposure | |
| **Which of the following symptoms did you experience post-vaccination?**  🞎Chills and fever 🞎Headache Anosmia 🞎 Sore throat 🞎 🞎 Exhaustion, fatigue  🞎 Dyspnea 🞎 Muscle sore Rhinorrhea 🞎 Ageusia 🞎 Cough 🞎  🞎 Nausea and vomiting 🞎 Other …………………. | |
| **History of MS worsening?** 🞎Yes 🞎No  **MS Relapse?** 🞎Yes 🞎No  **Worsening or new neurological symptoms?**  🞎Yes, what symptoms? ………… 🞎No | |
| **Underlying medical condition:**  🞎Obesity 🞎Cancer 🞎Diabetes 🞎Pregnancy 🞎Immunodeficiency  🞎 Cardiovascular diseases 🞎Asthma Autoimmune diseases 🞎  🞎 Other………………. | |
| **Laboratory technician section** Antibody Titer……………… | |

*No patient identification was recorded in the questionnaire.
